# Cytogenetic Investigation of Infertile Patients in Hungary: A 10-Year Retrospective Study

**DOI:** 10.3390/genes13112086

**Published:** 2022-11-10

**Authors:** Szilvia Andó, Katalin Koczok, Beáta Bessenyei, István Balogh, Anikó Ujfalusi

**Affiliations:** 1 Division of Clinical Genetics, Department of Laboratory Medicine, Faculty of Medicine, University of Debrecen, 4032 Debrecen, Hungary; 2 Department of Human Genetics, Faculty of Medicine, University of Debrecen, 4032 Debrecen, Hungary

**Keywords:** infertility, cytogenetics, chromosomal aberrations, single-cell translocations

## Abstract

Chromosome abnormalities play a crucial role in reproductive failure. The presence of numerical or structural aberrations may induce recurrent pregnancy loss or primary infertility. The main purpose of our study was to determine the types and frequency of chromosomal aberrations in infertile patients and to compare the frequency of structural aberrations to a control group. Karyotyping was performed in 1489 men and 780 women diagnosed with reproductive failure between 2010 and 2020. The control group included 869 male and 1160 female patients having cytogenetic evaluations for reasons other than infertility. Sex chromosomal aberrations were detected in 33/1489 (2.22%) infertile men and 3/780 (0.38%) infertile women. Structural abnormalities (e.g., translocation, inversion) were observed in 89/1489 (5.98%) infertile men and 58/780 (7.44%) infertile women. The control population showed structural chromosomal abnormalities in 27/869 (3.11%) men and 39/1160 (3.36%) women. There were significant differences in the prevalence of single-cell translocations between infertile individuals (males: 3.5%; females: 3.46%) and control patients (males: 0.46%; females: 0.7%). In summary, this is the first report of cytogenetic alterations in infertile patients in Hungary. The types of chromosomal abnormalities were comparable to previously published data. The prevalence of less-studied single-cell translocations was significantly higher in infertile patients than in the control population, supporting an earlier suggestion that these aberrations may be causally related to infertility.

## 1. Introduction

Infertility is a condition characterized by the failure to conceive after 12 months of regular, unprotected sexual intercourse [1,2,3,4]. It affects about 10–15% of couples in the Western countries [1,2]. There are several causes that lead to infertility. The most common factor is sperm abnormality in men and ovulation dysfunction and tubar pathology in women [1]. Furthermore, infertility can also be due to hormonal, immunological, genetical, psychological and other causes [3]. However, the cause of infertility remains unclear in a significant proportion (about 15–20%) of all cases [3,4].

Chromosomal abnormalities play an important role in reproductive failure. These can be numerical or structural and can affect sex chromosomes or autosomes. In general, autosomal numerical aberrations are not compatible with life, except in rare mosaic cases and in the well-characterized trisomy syndromes (Down (+21), Edwards (+13), Patau (+18)). These chromosome changes, nonetheless, are not characteristic causes for infertility. However, alterations in the number of sex chromosomes are frequently detected in reproductive failure. The most common structural changes are translocations, inversions, deletions and duplications [5].

In males, the most common (1:2000) sex chromosomal numerical aberration is the presence of one or more extra chromosome X, resulting in 47,XXY or 48,XXXY karyotype [6,7] as in Klinefelter syndrome characterized by a specific phenotype including infertility. In mosaic cases (47,XXY/46,XY), the phenotypic disturbances are milder with intact fertility [7]. A much rarer chromosomal abnormality is the 46,XX karyotype with male phenotype or structural Y aberration [6]. In women, the most common form of infertility is caused by numerical chromosome abnormality, where the loss of one sex chromosome results in Turner syndrome (45,X) that also has mosaic forms (e.g., 45,X/46,XX) [6,8]. Additionally, 47,XXX karyotype with 45,X mosaicism or 46,XY gonadal dysgenesis are causative alterations in female infertility [6].

Implicated in both sexes, autosomal abnormalities can be translocations (Robertsonian or reciprocal), inversions or other structural abnormalities [6]. Balanced autosomal translocations are frequent causes of infertility, where the carriers of the aberrations generally have a normal phenotype, and the effect on the pregnancy outcome is probably due to the conception of gametes with unbalanced combination of parental rearranged chromosomes [9].

In general, with an incidence of 1–3% in the healthy population, the most common type of inversion is the pericentric inversion of chromosome 9 (inv(9)), where the breakpoints are variable with the most common variant inv(9)(p11q12)/inv(9)(p11q13) [10,11]. Several authors have studied the clinical consequences of this structural variant, but the results remain contradictory. Some authors suggest that inv(9) is more common in patients with reproductive failure [12,13], while others consider it as a normal variant [14,15].

Single Cell Translocation (SCT) is described as an isolated metaphase of a sporadic cell with chromosomal translocation in an individual with an otherwise normal chromosomal constitution. SCTs were first described in the 1980s, but only a few subsequent studies reported SCTs as a possible cause of reproductive failure [16,17,18,19,20]. The most frequent form of this chromosomal rearrangement is the translocation between chromosomes 7 and 14 (t(7;14)), but the translocational breakpoints tend to be very heterogenous [16,21]. Early studies described a possible association between SCTs and multiple abortions, as well as the outcome of ICSI [19]. Although the probable cause of the origin of SCT and its clinical significance is not clearly understood, it may reflect chromosomal instability or may be a conseqence of the cell culture conditions [19].

Chromosomal aberrations are one of the principal genetic factors in infertility; therefore, constitutional karyotype analysis is essential in the diagnostic evaluation for identification of the underlying cause of reproductive failure. So far, no retrospective study of chromosomal abnormalities has been performed in Hungarian infertile individuals; therefore, the aim of our study was to evaluate the type and frequency of the cytogenetic abnormalities in infertile patients who were referred to our laboratory and to compare the frequency of structural chromosomal aberrations to gender-matched controls.

The study population included patients with different forms of infertility: primary, secondary and recurrent miscarriage. In routine practice, the evaluation of chromosomal abnormalities in individuals with reproductive failure helps the genetic counselors in risk evaluation and fertility experts and obstetricians in selecting the most appropriate management and treatment for infertile patients.

## 2. Materials and Methods

In this retrospective study, we analysed the cytogenetic results of 1489 infertile men (ages: 21–54 years) and 780 women (ages: 25–45 years) who were referred to the cytogenetic unit at the Department of Laboratory Medicine, University of Debrecen in Hungary between January 2010 and December 2020.

The control group consisted of 869 males and 1160 females referred to the cytogenetic laboratory due to indications other than infertility (e.g., congenital abnormality, developmental delay, carrier testing, etc.) and had normal karyotype. Individuals with pathogenic chromosomal abnormormalities (e.g., aneuploidy, unbalanced translocations) were excluded. The control patients were examined in the same period.

All participants gave informed consent to genetic testing according to national regulations. The laboratory is approved by The National Public Health and Medical Officer Service (Approval number: 094025024).

Conventional chromosome analysis was performed according to standard protocol. A heparinized peripheral blood sample was collected from the patients, and chromosomal analysis was carried out on phytohemagglutinin (PHA) stimulated lymphocyte cultures. At least ten Giemsa-banded metaphases were analysed using Lucia Karyo software. In cases with single-cell translocations, we counted additional metaphases (altogether 30) to exclude (or confirm) real mosaicism according to the recommendation of the European guidelines for constitutional cytogenomic analysis [22]. This number of analysed metaphases permits the detection of a mosaicism rate ≥8% at a 0.90 confidence level and ≥15% at a 0.99 confidence level [23]. According to our experience, the single-cell abnormality was confirmed in all cases by the analysis of 30 metaphases. The karyotypes were recorded according to the actual version of the International System for Human Cytogenomic Nomenclature (ISCN).

Statistical analysis was performed using GraphPad Prism Version 9.1.2. Categorical parameters were analyzed using the χ^2^ test.

## 3. Results

**Figure 1:**
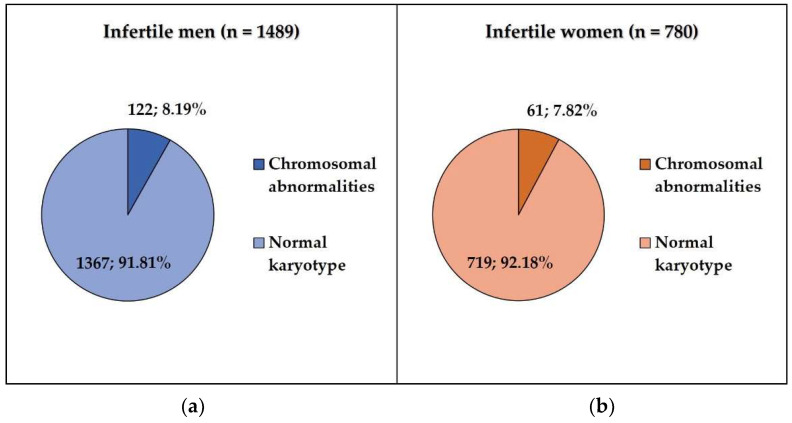
(**a**) The frequency of chromosomal abnormalities in infertile men; (**b**) the frequency of chromosomal abnormalities in infertile women.

**Figure 2:**
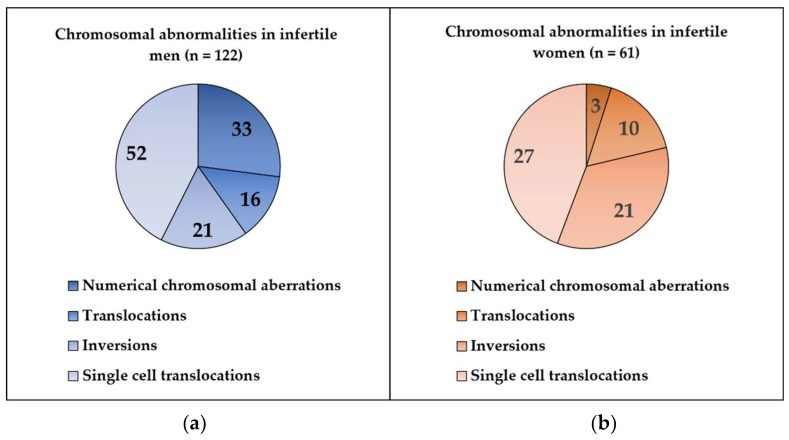
(**a**) Type of the chromosomal abnormalities in infertile men; (**b**) type of chromosomal abnormalities in infertile women.

In this retrospective study, we analyzed the cytogenetic results of 1489 men (ages: 21–54 years) and 780 women (ages: 25–45 years) who were referred to constitutional chromosome analysis in the cytogenetic unit because of different forms of reproductive failure (primary or secondary infertility, recurrent miscarriage). Chromosomal abnormalities were detected in 8% of all cases, while 92% showed normal karyotype. In the studied infertile men, we found 122 cases (8.19%) with chromosomal abnormalities, and 1367 (91.98%) had normal karyotype. Among the studied infertile women, 61/780 (7.82%) cases showed chromosomal abnormalities, and 719/780 (92.18%) had normal karyotype (Figure 1). Numerical chromosome alterations affected only sex chromosomes, while structural aberrations (reciprocal balanced translocations, inversions) involved both autosomes and sex chromosomes. Cytogenetically visible heterochromatic variants (variant size) and satellite size are not routinely included in the patients’ cytogenetic reports; therefore, these data could not be collected. The most frequent type of chromosome abnormality was single-cell translocation in both infertile groups (Figure 2).

### 3.1. Infertile Men vs. Control Patients

#### 3.1.1. Numerical Chromosomal Aberrations

A total of 33 (2.22%) of 1489 infertile men showed numerical sex chromosomal aberration. In this group, the most frequent karyotype was 47,XXY, observed in 28 patients. Four patients had mosaic karyotype: 47,XXY/46,XY (*n* = 3); 48,XXXY/47,XXY (*n* = 1). One male patient’s karyotype was 46,XX (Table 1).

#### 3.1.2. Structural Chromosomal Aberrations

In our study, 89/1489 (5.98%) men showed structural chromosomal aberrations. Balanced translocation was found in 16 (1.07%) cases. The most frequent type was the Robertsonian translocation t(13;14)(q10;q10) (*n* = 4).

The most common chromosomal inversion was the pericentric inversion of chromosome 9, with a frequency of 1.07% (16/1489) in the infertile male group. The incidence of this variant was 2.65% (23/869 male) in the control population. Other types of inversions are shown in Table 1.

**Figure 3:**
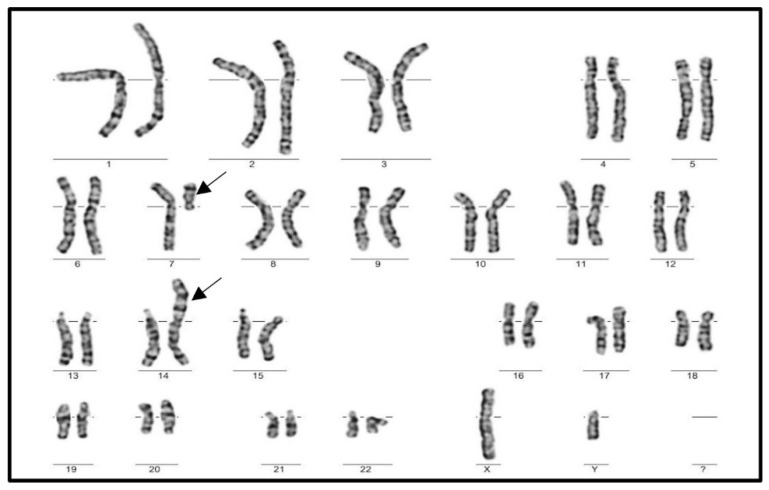
Karyogram of a male patient with the most frequent SCT, t(7;14).

**Figure 4:**
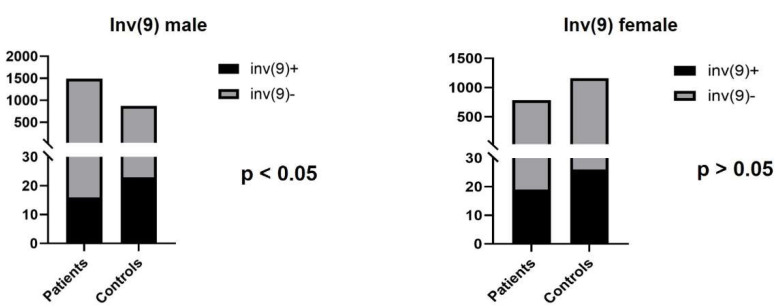
The number of infertile patients and control individuals with inv(9).

**Figure 5:**
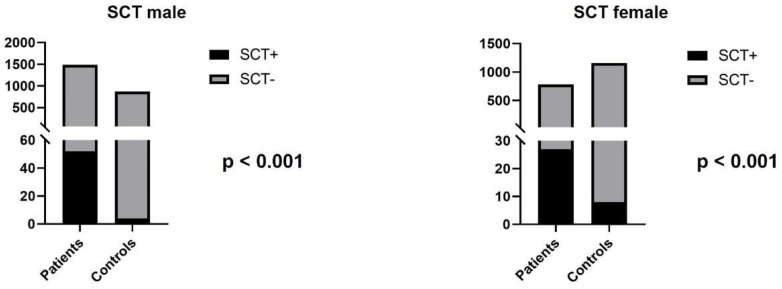
The number of SCTs in infertile patients and control individuals.

Single cell translocation was identified in 52 cases (3.49%) in infertile men (Figure 2). The t(7;14) was the most frequent translocation (12/52) (Figure 3), while all other translocations affected autosomes randomly. The incidence of the single-cell translocation was 0.46% (4/869 male) in the control population (Table 1 Figure 4 and Figure 5).

### 3.2. Infertile vs. Control Women

#### 3.2.1. Numerical Chromosomal Aberrations

In infertile females, 3 of the 780 (0.38%) had sex chromosomal abnormalities. Numerical alteration of chromosome X was detected in mosaic form in two cases (45,X/46,XX), and one female patient presented with 46,XY karyotype (Table 2).

#### 3.2.2. Structural Chromosomal Aberrations

In the infertile female group 58/780 (7.43%), cases showed structural chromosomal aberrations. Balanced translocation was found in 10/780 (1.28%) patients, affecting different autosomes.

Among the infertile females, two types of chromosomal inversions were detected: inv(9) (*n* = 19; 2.44%) and inv(10) (*n* = 2; 0.26%). In the control group, the most common inversion was also inv(9) (*n* = 26; 2.24%) (Figure 4).

Single cell translocation was identified in 27 cases (3.46%). The t(7;14) translocation was found in 2/27 cases. In the control population, the incidence of the single-cell translocation was 0.69% (8/1160 female) (Table 2 and Table 3 and Figure 5).

## 4. Discussion

In the present study, we evaluated the type and frequency of cytogenetic abnormalities in infertile male and female patients and compared the frequency of structural chromosomal aberrations in gender-matched control individuals.

The results showed that the prevalence of numerical and structural chromosomal abnormalities was 122/1489 (8.19%) in infertile men and 61/780 (7.82%) in infertile women. A total of 33 of the 1489 infertile men (2.22%) showed sex chromosomal aberrations, and 89/1489 (5.98%) had structural abnormalities. Three of the 780 infertile women (0.38%) showed sex chromosomal alterations, and 58 (7.44%) had structural abnormalities. Non-pathological structural chromosomal abnormalities were detected in 27/869 (3.11%) control male cases and 39/1160 (3.36%) control female samples. Our results are hard to compare to previously published data due to the types and definitions of chromosomal alterations reported by others [11,24,25,26,27,28]. Most of the studies investigating cytogenetic cause of reproductive failure also included heterochromatic polymorphisms among chromosomal aberrations [11,24,25], while others did not include these [26,27,28]. Generally, most reports provided no clarification for the inclusion or exclusion of polymorphic variants in their study. Gada et al. did not include inv(9) and inv(Y), 1qh, 9qh, 16qh and Yqh variants, heterochromatic polymorphisms (15p and 22p) and large satellites, as they considered these to have no clinical significance [26].

According to the European cytogenetic guideline for constitutional cytogenomic analysis, the polymorphic variants that have been previously reported as harmless should be excluded from the reports (to avoid confusion for the non-specialists). Following this recommendation, in our routine practice the cytogenetically visible heterochromatic variants (variant size) and satellite size are not included in the cytogenetic report; therefore, our study did not contain any data about their frequency.

There are very few published data about single-cell translocations; their frequency and impact in infertility has not been extensively studied [16,20].

In our study, the most frequent abnormal karyotype affecting sex chromosomes was the 47,XXY (Klinefelter syndrome) in infertile male patients and 45,X/46,XX (mosaic Turner syndrome) in case of infertile women. Based on literature data, the prevalence of Klinefelter syndrome in patients with reproductive failure is between 3–4% [7]. In our infertile male cohort, the frequency was lower (2.15%). The prevalence of numerical X chromosomal mosaicism was 0.26% in our infertile women, whereas Gekas et al. reported a prevalence of 2.77% [29]. In their study, Gekas et al. used a 3% cut-off limit for detemination of real mosaicism, while in our study we defined the cut-off for low-level mosaicism at 10%. This difference in the cut-off values can explain the lower occurence of mosaic numerical X chromosomal aberrations in infertile women in our study (0.26%) compared to their data (2.77%).

There are no evidence-based data regarding the appropriate cut-off limit in case of low-level mosaicism. Generally, in the literature, the cut-off limit of low-level mosaicism ranges between 4% and 10%, but according to the European Cytogeneticists Association guidelines, the limit should be 10% and 15% for low-level and true-mosaicism, respectively. The significance of low-level mosaicism of 45,X/46,XX is unclear, particularly in cases with low aneuploid cell counts (<6–10% of cells). As a result of these studies, it is generally considered that less than 10% cells with X aneuploidy are without reproductive significance [30,31]. In line with this data, we did not include the X mosaic cases with <10% of aneuploid cells.

We found two infertile patients (one male with 46,XX and one female with 46,XY karyotype) who had disorders of sex development (DSD), a group of conditions in which chromosomal, gonadal or phenotypic sex is atypical. In these conditions, the patients usually have fertility problem. The estimated prevalence of DSD is 1/20.000~25.000 [32].

The incidence of autosomal translocations was 1.07% in infertile men and 1.28% in infertile women, while in a comparable published study it was 1.59% (10/629) for men and 2.55% (16/629) for women [11].

Because of the controversy in literature data concerning the frequency of inversions, especially the inv(9), and single-cell translocations in infertile individuals, our aim was to study the occurrence of these structural aberrations in our cohort. Inversion is one of the most common structural chromosomal balanced rearrangements where a two-break event occurs followed by a 180-degree turn of the segment and reinsertion at its original breakpoints. Carriers of inversion usually have a normal phenotype without DNA loss or gain at the breakpoints. However, in balanced inversion carriers reproductive failure (higher rates of miscarriage, diminished fertility), and offspring with congenital anomalies have also been described [33]. The mechanism of chromosomal abnormality affecting gametogenesis and the resulting reproductive failure can be the following: (i) numerical chromosomal abnormalities in the gametes by interchromosomal effect (ICE), (ii) the inverted chromosome region can cause synaptic and recombinational problems during meiosis depending of the length of the inversion: the larger the inversion, the more frequently recombinants are observable in the gametes. (iii) DNA fragmentation in spermatozoa and activation of apoptosis, and (iv) alteration of gene function at the breakpoints [34,35,36]. However, Young et al. could not confirm the presence of increased aneuploid sperm for chromosomes that were not involved in the inversion carriers [37]. Therefore, the exact mechanism of chromosomal abnormality affecting gametogenesis in carriers of chromosomal inversion requires further study.

The pericentric inversion of chromosome 9 is the most frequently studied inversion, although the results are not concordant according to its clinical consequences. Several studies suggest that inv(9) is more common in patients with reproductive failure [12,13], while other researchers did not find any causal relationship [14,15]. Mozdarani et al. found one male (0.33%) and 14 female cases (4.69%) with inv(9) among 601 infertile patients. In another study that examined 900 couples with reproductive failure, the incidence of inv(9) was 2.73% in males and 1.92% in females [10,14]. In our study, inv(9) was the most frequent type of chromosomal inversions (1.07% in men; 2.44% in women). According to our data, the prevalence of inv(9) was significantly lower compared to the control group in infertile male patients (1.07% vs. 2.65%; *p* < 0.05) and was similar in the female test and control groups (2.44% vs. 2.24%). These results suggest that the inv(9) may be a benign chromosomal variant and does not show association with reproductive failure. As there is no conclusive evidence for the pathogenicity of this rearrangement, further clinical and molecular studies would be helpful to clarify the role of inv(9) in infertility [38].

Balanced reciprocal translocations are common structural aberrations in reproductive failure. They are typically benign, without any phenotypic consequences in carriers. However, meiosis in germ cells with balanced translocations may result in meiotic arrest and subsequent infertility or unbalanced gametes, resulting in miscarriage or unbalanced offspring. Most reciprocal translocations are unique regarding their breakpoints [39]. According to previously published data, the chromosomal breakpoints involved in constitutional balanced chromosomal rearrangements (e.g., translocations, inversions) appear in a non-random distribution along the human chromosomes [40]. Based on the study of Manvelyan et al., ~88% of chromosomal break-events arose in GTG-light bands, 21% co-localized with intrachromosomal telomeric-like sequences (ITS), 35.8% were at or near the Mariner transposon-like elements (MTLE), and at least 45% could have had a correlation with fragile sites (FS) [41]. The importance of fragile sites in balanced reciprocal translocation was supported by Liehr et al. reporting cytogenetic co-localization of fragile sites in ~71% of the studied 529 break-events in 251 cases with balanced chromosomal rearrangements [36].

A special type of balanced translocations are the single-cell translocations (SCTs) that were described in the early 1980s and related to fertility problems. In our study, we observed SCTs in a large proportion of structural aberrations in infertile patients (52/122 in men; 27/61 in women). SCTs are fairly understudied, with only a couple of research articles published. Although the translocation breakpoints are very different in SCTs, translocation between chromosome 7 and 14 (t(7;14)) is more frequent than others [16,21]. In 1978, Zech and Haglund reported 12 cases of SCTs between chromosome 7 and 14 in a total of 5500 metaphases from PHA-stimulated lymphocytes of 45 patients. There were three types of translocation according to their breakpoints: t(7;14)(qter;q12.3) (*n* = 6), t(7;14)(pter;q22) or (p13;q11.2) (*n* = 5) and t(7;14)(q32;qter) (*n* = 1). The authors suggested that the PHA might be responsible for producing this sporadic, single-cell translocation [42]. Wallace et al. in 1984 reported 12 sporadic t(7;14) in 661 metaphases from 10 individuals investigated in the routine cytogenetic series, and two different types of translocation breakpoints were observed: t(7;14)(p13;q11.2) and t(7;14)(q3?2–4;q11.2) [43]. Dewald et al. identified 83 individuals from 11 915 patients and 37 normal controls with sporadic t(7;14) SCT. Among the 83 cases, 82 were observed in lymphocyte culture, and one was in a fibroblast culture. Four types of breakpoints were described, but in 81/83 cases, the translocations were t(7;14)(q34;q11) (*n* = 42) or t(7;14)(p13;q11) (*n* = 39). None of these two types of translocations were detected in fibroblast, amniocyte, bone marrow or unstimulated peripheral blood metaphases. They found that this type of translocation does not occure more often during any month or season of the year and does not seem to be influenced by sex and age. The authors suggested that t(7;14) SCTs may be specifically associated with T-lymphocytes, that are overrepresented as dividing cells in the PHA-stimulated culture. The opportunity of such a sporadic translocations in T-cells may be further increased by the possibility that bands 7p13, 7q34 and 14q11 get closer to each other in interphase. These chromosomal loci contain transcriptionally active genes (α and β chain of T-cell receptors) that are involved in normal physiological processes of T-cells. Because of the constitutive activation of these genes, they are more vulnerable to DNA damage or breaks under culture condition [44].

Some authors found that SCT has a higher incidence in couples with multiple miscarriages. In a previously published study, 21 SCT were found in 555 patients, and there was a significantly higher number of SCTs among individuals with habitual abortions compared to patients with other referral reasons, and furthermore the most frequent translocation was the t(7;14) [17]. In another publication, 389 patients with adverse obstetric history were investigated, and single-cell abnormality was found in 28 individuals [16]. In our study, the prevalence of SCT was significantly higher in infertile patients (3.49% vs. 0.46% in men and 3.46% vs. 0.69% in women; *p* < 0.001). The most frequent type of these translocations was t(7;14) in accordance with the literature data. The remaining SCTs did show random involvement of chromosomes and breakpoints. The pathogenic role of these rearrangements is still unclear, and further studies are needed to reveal their clinical significance. This type of aberration is not reported in the less-studied germ cells; therefore, we cannot conclude that they are related to infertility, but our results can help the routine SCT-related work of cytogenetic laboratories.

## 5. Conclusions

In summary, this is the first, comprehensive, large-scale study in Hungary investigating the cytogenetic background of individuals with reproductive failure. The types of chromosomal aberrations were similar to the previously published data, while there were statistically significant differences in the prevalence of certain structural aberrations, such as inv(9) and SCTs. According to our results, the prevalence of inv(9) was significantly lower in infertile men compared to the control population. We did not observe significant difference in the frequency of inv(9) in female patients. These results suggest that the inv(9) may be a benign chromosomal variant and does not show association with reproductive failure. In contrast, SCTs were detected in a higher percent of infertile patients, confirming the earlier findings. Further studies are needed to clarify the clinical consequences of SCTs in reproductive failure.

## Figures and Tables

**Table 1:** Chromosomal abnormalities in infertile male patients and control group.

Type of Aberrations		Karyotype	No. of Cases	%
Infertile Patients (*n* = 1489)				
DSD (46,XX male)		46,XX	1	0.07
Numerical chromosomal aberrations		47,XXY	28	1.88
		47,XXY[3]/46,XY[12] (*n* = 1); 47,XXY[10]/46,XY[5] (*n* = 1); 47,XXY[14]/46,XY[1] (*n* = 1)	3	0.21
		48,XXXY[1]/47,XXY[14]	1	0.07
Structural chromosomal aberrations	Translocations (*n* = 16; 1.07%)	45,XY,rob(13;14)(q10;q10)	4	0.28
		45,XY,rob(14;21)(q10;q10)	1	0.07
		46,XY,t(2;5)(p21?;q31)	1	0.07
		46,XY,t(3;6)(q21;q23)	1	0.07
		46,XY,t(5;14)(q31;q11.2)	1	0.07
		46,XY,t(7;20)(p15;p13)	1	0.07
		46,XY,t(8;15)(p21;q26)	1	0.07
		46,XY,t(10;22)(q26;q13)	1	0.07
		46,XY,t(11;22)(q23;q11)	1	0.07
		46,Y,t(X;3)(p1?1.2;q2?7)	1	0.07
		46,XY,t(Y;3)(q12;q22?)	1	0.07
		46,XY,t(11;22)(q23.3;q11.2)[8]/46,XY[7]	1	0.07
		46,XY,t(14;19)(q24;q13)[5]/46,XY[10]	1	0.07
	Inversions (*n* = 21; 1.41%)	46,XY,inv(9)(p13q21) (*n* = 10); 46,XY,inv(9)(p12q13) (*n* = 6)	16	1.07
		46,XY,inv(5)(q13q35) (*n* = 1); 46,XY,inv(5)(q31q33) (*n* = 1)	2	0.14
		46,XY,inv(16)(q11.2q22)	1	0.07
		46,XY,inv(10)(p11.2q21.2)	1	0.07
		46,XY,inv(12)(p13q12)	1	0.07
	Single cell translocations	(Table 3.)	52	3.5
Control group (*n* = 869)				
Structural chromosomal aberrations	Inversions	46,XY,inv(9)(p12q13) (*n* = 12); 46,XY,inv(9)(p13q21) (*n* = 11)	23	2.65
	Single cell translocations	(Table 3.)	4	0.46

**Table 2:** Chromosomal abnormalities in infertile female patients and controls.

Type of Aberrations		Karyotype	No. of Cases	%
Infertile Patients (*n* = 780)				
DSD (46,XY female)		46,XY	1	0.13
Numerical chromosomal aberrations		45,X[2]/46,XX[13] (*n* = 1); 45,X[1]/46,XX[14] (*n* = 1)	2	0.26
Structural chromosomal aberrations	Translocations (*n* = 10; 1.28%)	45,XX,rob(13;14)(q10;q10)	1	0.13
		45,XX,rob(13;22)(q10;q10)	1	0.13
		45,XX,rob(14;15)(q10;q10)	1	0.13
		45,XX,rob(14;21)(q10;q10)	1	0.13
		46,XX,t(1;4)(q?;q?)	1	0.13
		46,XX,t(1;16)(p36;p13)?	1	0.13
		46,XX,t(2;6)(p2?3;q2?1)	1	0.13
		46,XX,t(2;10)(p11?;p11?)	1	0.13
		46,XX,t(3;11)(p2?3;q23)	1	0.13
		46,XX,t(9;20)(p?;p?)	1	0.13
	Inversions (*n* = 21; 2.69%)	46,XX,inv(9)(p12q13) (*n* = 12); 46,XX,inv(9)(p13q21) (*n* = 7)	19	2.44
		46,XX,inv(10)(p11q21)	2	0.26
	Single cell translocations	(Table 3.)	27	3.46
Control group (*n* = 1160)				
Structural chromosomal aberrations	Inversions (*n* = 31; 2.67%)	46,XX,inv(9)(p13q21) (*n* = 15); 46,XX,inv(9)(p12q13) (*n* = 11);	26	2.24
		46,XX,inv(10)(p11.2q22.1)	2	0.17
		46,XX,inv(1)(p13q21)	1	0.09
		46,XX,inv(5)(q11.2q22)	1	0.09
		46,XX,inv(13)(q12q14.?1)	1	0.09
	Single cell translocations	(Table 3.)	8	0.7

**Table 3:** Types of SCTs in infertile and control patients.

Infertile Patients (*n* = 2269)			
t(7;14)(q34;q11.2) (*n* = 8; 0.35%)	t(5;9)(p15;p11) (*n* = 1; 0.04%)	t(2;8)(q33;q22) (*n* = 1; 0.04%)	t(7;17)(q32;q21) (*n* = 1; 0.04%)
t(7;14)(q34;q11.2)/t(1;17)(q42;q25) (*n* = 1; 0.04%)	t(5;9)(q35;q13) (*n* = 1; 0.04%)	t(3,5)(q21;q35) (*n* = 1; 0.04%)	t(7;18)(p22;q21) (*n* = 1; 0.04%)
t(7;14)(p15.3;q13) (*n* = 2; 0.09%)	t(6;6)(q21;p21) (*n* = 1; 0.04%)	t(3;7)(q12;p15) (*n* = 1; 0.04%)	t(7;22)(?;?) (*n* = 1; 0.04%)
t(7;14)(q10;q10) (*n* = 1; 0.04%)	t(6;6)(q21;p25) (*n* = 1; 0.04%)	t(3;7)(q23;q22) (*n* = 1; 0.04%)	t(8;11)(p23.3;p15.5) (*n* = 1; 0.04%)
t(7;14)(q11;p11) (*n* = 1; 0.04%)	t(7;12)(p15;q13) (*n* = 1; 0.04%)	t(3;10)(p13;q26) (*n* = 1; 0.04%)	t(8;14)(q24;q24) (*n* = 1; 0.04%)
t(7,14)(q32;q32) (*n* = 1; 0.04%)	t(7;12)(q36;q22) (*n* = 1; 0.04%)	t(3;13)(q27;q14) (*n* = 1; 0.04%)	t(9;13)(p13;q22) (*n* = 1; 0.04%)
t(7;14)(q3?5;q11) (*n* = 1; 0.04%)	t(8;16)(p12;q24) (*n* = 1; 0.04%)	t(4;6)(q27;q23) (*n* = 1; 0.04%)	t(9;20)(?;?) (*n* = 1; 0.04%)
t(7;14)(q3?6;q13) (*n* = 1; 0.04%)	t(8;16)(p21;q13) (*n* = 1; 0.04%)	t(4;8)(p14;q22) (*n* = 1; 0.04%)	t(10;12)(q22;q13) (*n* = 1; 0.04%)
t(7;14)(q3?6;q13) (*n* = 1; 0.04%)	t(10;11)(p13;q25) (*n* = 1; 0.04%)	t(4;11)(p16;q13) (*n* = 1; 0.04%)	t(10;16)(q22;q22) (*n* = 1; 0.04%)
t(7;14)(p13;q11) (*n* = 1; 0.04%)	t(10;11)(q22;p15) (*n* = 1; 0.04%)	t(4;12)(q21;p13) (*n* = 1; 0.04%)	t(10;18)(q24;q23) (*n* = 1; 0.04%)
t(1;2)(p22;p13) (*n* = 1; 0.04%)	t(1;6)(p22;p21) (*n* = 1; 0.04%)	t(4;17)(q24;q21) (*n* = 1; 0.04%)	t(11;12)(q21;q13) (*n* = 1; 0.04%)
t(1;2)(p22;p21) (*n* = 1; 0.04%)	t(1;7)(p32;q32) (*n* = 1; 0.04%)	t(5;12)(q31;q13) (*n* = 1; 0.04%)	t(11;13)(q13;q21) (*n* = 1; 0.04%)
t(1;5)(q32;p15.2) (*n* = 1; 0.04%)	t(1;10)(p36;p11) (*n* = 1; 0.04%)	t(6;12)(q27;q13.1) (*n* = 1; 0.04%)	t(14;16)(q24;q24) (*n* = 1; 0.04%)
t(1;5)(p22;q31) (*n* = 1; 0.04%)	t(1;11)(p32;q23) (*n* = 1; 0.04%)	t(6;13)(p11;q21.2) (*n* = 1; 0.04%)	t(14;20)(p11;p11) (*n* = 1; 0.04%)
t(2;7)(q35;p22) (*n* = 1; 0.04%)	t(1;13)(q32;q22) (*n* = 1; 0.04%)	t(6;20)(q13;q13) (*n* = 1; 0.04%)	t(X;1)(q32;p22) (*n* = 1; 0.04%)
t(2;7)(q33;q33)/t(2;12)(q33;q13.1) (*n* = 1; 0.04%)	t(1;14)(p36;q13)/t(15;18)(q13;q11) (*n* = 1; 0.04%)	t(7;8)(q32;q24) (*n* = 1; 0.04%)	t(X;11)(q13;p15) (*n* = 1; 0.04%)
t(2;11)(p21?;p15?) (*n* = 1; 0.04%)	t(1;18)(q32;q11) (*n* = 1; 0.04%)	t(7;10)(p13;q11) (*n* = 1; 0.04%)	t(Y;16)(q12;q13.3) (*n* = 1; 0.04%)
t(2;11)(p25;p11) (*n* = 1; 0.04%)	t(1;22)(q23;q13) (*n* = 1; 0.04%)	t(7;16)(q11;q12) (*n* = 1; 0.04%)	
Control patients (*n* = 2029)			
t(7;14)(q11;p11) (*n* = 1; 0.05%)	t(7;14)(q34;q11) (*n* = 1; 0.05%)	t(14;19)(q11;q13) (*n* = 1; 0.05%)	t(7;20)(?;?) (*n* = 1; 0.05%)
t(7;14)(q11.2;p11) (*n* = 1; 0.05%)	t(7;14)(q34;q11)/t(3;10)(q27;q21) (*n* = 1; 0.05%)	t(14;19)(q11;p13) (*n* = 1; 0.05%)	t(7;22)(p32;q11) (*n* = 1; 0.05%)
t(7;14)(p21;q22) (*n* = 1; 0.05%)	t(7;14)(q36;q13) (*n* = 1; 0.05%)	t(7;10)(q36;q11) (*n* = 1; 0.05%)	t(9;12)(?;?) (*n* = 1; 0.05%)

## Data Availability

The data presented in this study are available on request from the corresponding author.
